# Biallelic variants in *ADARB1*, encoding a dsRNA-specific adenosine deaminase, cause a severe developmental and epileptic encephalopathy

**DOI:** 10.1136/jmedgenet-2020-107048

**Published:** 2020-07-27

**Authors:** Reza Maroofian, Jiří Sedmík, Neda Mazaheri, Marcello Scala, Maha S Zaki, Liam P Keegan, Reza Azizimalamiri, Mahmoud Issa, Gholamreza Shariati, Alireza Sedaghat, Christian Beetz, Peter Bauer, Hamid Galehdari, Mary A O’Connell, Henry Houlden

**Affiliations:** 1 Department of Neuromuscular Disorders, UCL Queen Square Institute of Neurology, London, WC1N 3BG, UK; 2 CEITEC, Masaryk University, Kamenice 735/5, A35, Brno 62500, Czech Republic; 3 Department of Genetics, Faculty of Science, Shahid Chamran University of Ahvaz, Ahvaz, Iran; 4 Department of Neurosciences, Rehabilitation, Ophthalmology, Genetics, Maternal and Child Health, Università degli Studi di Genova, Genova, Italy; 5 Clinical Genetics Department, Human Genetics and Genome Research Division, National Research Centre, Cairo, Egypt; 6 Department of Paediatric Neurology, Golestan, Medical, Educational, and Research Center, Ahvaz Jundishapur University of Medical Sciences, Behbahan, Iran; 7 Department of Medical Genetics, Faculty of Medicine, Ahvaz Jundishapur University of Medical Sciences, Behbahan, Iran; 8 Health Research Institute, Diabetes Research Center, Ahvaz Jundishapur University of medical Sciences, Ahvaz, Iran; 9 CENTOGENE AG, Rostock, Germany

**Keywords:** epilepsy, mutation, missense, DNA, sequence analysis, nervous system diseases

## Abstract

**Background:**

Adenosine-to-inosine RNA editing is a co-transcriptional/post-transcriptional modification of double-stranded RNA, catalysed by one of two active adenosine deaminases acting on RNA (ADARs), ADAR1 and ADAR2. *ADARB1* encodes the enzyme ADAR2 that is highly expressed in the brain and essential to modulate the function of glutamate and serotonin receptors. Impaired ADAR2 editing causes early onset progressive epilepsy and premature death in mice. In humans, ADAR2 dysfunction has been very recently linked to a neurodevelopmental disorder with microcephaly and epilepsy in four unrelated subjects.

**Methods:**

We studied three children from two consanguineous families with severe developmental and epileptic encephalopathy (DEE) through detailed physical and instrumental examinations. Exome sequencing (ES) was used to identify *ADARB1* mutations as the underlying genetic cause and in vitro assays with transiently transfected cells were performed to ascertain the impact on ADAR2 enzymatic activity and splicing.

**Results:**

All patients showed global developmental delay, intractable early infantile-onset seizures, microcephaly, severe-to-profound intellectual disability, axial hypotonia and progressive appendicular spasticity. ES revealed the novel missense c.1889G>A, p.(Arg630Gln) and deletion c.1245_1247+1 del, p.(Leu415PhefsTer14) variants in *ADARB1* (NM_015833.4). The p.(Leu415PhefsTer14) variant leads to incorrect splicing resulting in frameshift with a premature stop codon and loss of enzyme function. In vitro RNA editing assays showed that the p.(Arg630Gln) variant resulted in a severe impairment of ADAR2 enzymatic activity.

**Conclusion:**

In conclusion, these data support the pathogenic role of biallelic *ADARB1* variants as the cause of a distinctive form of DEE, reinforcing the importance of RNA editing in brain function and development.

## Introduction

One of the most widespread forms of RNA modification in the human transcriptome is the conversion of adenosine-to-inosine (A-to-I), mediated by active members of the adenosine deaminases acting on RNA (ADAR) family of enzymes. ADARs are highly conserved across vertebrates and invertebrates, and are essential for normal development.^1 2^ Mammals have three ADAR proteins: ADAR1 (*ADAR*), ADAR2 (*ADARB1*), which catalyse the A-to-I editing in double-stranded RNA (dsRNA) and ADAR3 (*ADARB2*), which is inactive but still binds to dsRNA. The editing and RNA-binding activities of ADARs affect RNA processing, RNA stability, and can even lead to recoding of open reading frames, since inosine in a codon is decoded as guanosine. The expression of ADARs varies across development and tissues in mammals.^3^

ADAR1 is widely expressed throughout the body and is the most highly expressed ADAR outside the central nervous system (CNS). Interestingly, both biallelic and monoallelic ADAR1 mutations resulting in altered editing have been associated with autoimmune conditions such as Aicardi-Goutières syndrome, dyschromatosis symmetrica hereditaria and bilateral striatal necrosis.^4–6^

ADAR2 and ADAR3 are most highly expressed in CNS, with more restricted expression in other tissues. ADAR2 is essential for recoding of brain pre-mRNAs, modulating the function of glutamate and serotonin receptors through the recoding of the transcripts of their subunits.^1 7 8^ In fact, impaired ADAR2 editing causes early onset progressive epilepsy and premature death in mice.^9^ In humans, biallelic *ADARB1* variants have been very recently associated with a severe developmental and epileptic encephalopathy (DEE) characterised by global developmental delay (DD), severe-to-profound intellectual disability (ID), microcephaly, epilepsy and limb spasticity.^10^

## Methods

### Subjects and samples

In this study, we report three subjects from two unrelated consanguineous families with a similar severe DEE, in whom exome sequencing (ES) and homozygosity mapping were performed to investigate the underlying genetic cause.

### Exome sequencing

To investigate the genetic cause of the disease in these two families, ES of DNA from probands of both families was performed. DNA was extracted using standard procedures from peripheral blood leucocytes. For family 1, ES and data analysis were performed as previously described.^11^ For family 2, ES and analysis were conducted at CENTOGENE.^12^ The candidate variants were confirmed, and segregation analysis was performed by Sanger sequencing.

### Mutation analysis

In accordance with the recessive mode of inheritance in both families, priority was given to rare biallelic functional variants with allele frequency <0.001% in public databases, including the 1000 Genomes project, NHLBI Exome Variant Server, Complete Genomics 69, gnomAD, GME Variome and Iranome as well as the in-house database consisting of 12 000 exomes and CENTOGENE internal database. After applying the above filtering criteria, no plausible compound heterozygous or homozygous variants were identified in genes previously associated with neurological phenotypes. However, further ES data analysis revealed candidate variants in *ADARB1* in both families.

### Cell culture and plasmids

All the cell lines were obtained from the European Collection of Authenticated Cell Cultures, and were grown as monolayers at 37°C with 5% CO_2_. HEK 293T were cultured in Minimum Essential Medium (MEM) with Earle’s Salts (biosera) supplemented with non-essential amino acids (Sigma-Aldrich), 10% fetal calf serum (FCS) and penicillin/streptomycin (biosera). HeLa were cultured in Dulbecco’s Modified Eagle Medium High Glucose (biosera) supplemented with non-essential amino acids, 10% FCS and penicillin/streptomycin. SH-SY5Y were cultured in Ham’s F12 medium and MEM with Earle’s Salts (mixed 1:1, biosera) supplemented with non-essential amino acids, 15% FCS and penicillin/streptomycin. All the cell lines were grown as monolayers at 37°C with 5% CO_2_.

The FLAG-tagged ADAR2 expression vectors were generated by PCR mutagenesis from the respective wild-type plasmids as described previously.^10^ Mutagenic primers containing the p.(Arg630Gln) variant were:

Fw: 5’-CAGAAGCACAGCAGCCAGGGAAGGCCCCCAACTTCAGTGTCA-3’;

Rv: 5’-CTGGCTGCTGTGCTTCTGCATTGCTGATGCCACTGAGCAAAGGC-3’.

For the splicing assay, primers with *attB* cloning sites were designed to amplify a minigene that contains the region around *ADARB1* exons 4 and 5 from human gDNA. The PCR product was cloned into pDESTsplice destination vector using Gateway Cloning.^13^ The primers used for the cloning PCR were:

First step Fw: 5’-AAAAAGCAGGCTGCTGTGTTCTAGTTGTGG-3’;

First step Rv: 5’-AGAAAGCTGGGTAAGGGATATTAACACAGG-3’;

Second step Fw: 5’-GGGGACAAGTTTGTACAAAAAAGCAGGCT-3’;

Second step Rv: 5’-GGGGACCACTTTGTACAAGAAAGCTGGGT-3’.

The mutagenic primers used to introduce the c.1245_1247+1 del variant into the wild-type splicing reporter plasmid were:

Fw: 5’-GCTTTACTTTAAGTTTAGTAAACAAATAAGGACAGGAAG-3’;

Rv: 5’-CTAAACTTAAAGTAAAGCTCAAGTTGTGTATAAAGAAATC-3’.

### RNA editing assay and splicing assay

For the editing assay, HEK 293 T cells were co-transfected with two plasmids, the ADAR2 expression vector and the RNA editing substrate expression vector, and the RNA from transfected cells was isolated as previously described.^10^ RT-PCR was performed with RevertAid RT Kit (Thermo Fisher) according to the manufacturer’s instructions with 2.5 µM of oligo(dT)_18_ used as a primer. PCR products encompassing the *pri-mir-376a2* editing site +4 or the *Gria2* Q/R editing site were subjected to Sanger sequencing, and the peak heights at the editing sites were measured from the sequencing chromatograms. Guanosine peak (G) represents the edited transcripts and adenosine peak (A) the unedited ones. The editing activity was calculated as G/(A+G), and the activity of the wild-type protein was set as 100%. Statistical significance of differences between samples was determined by two-tailed t-tests. The primers used for the editing assay PCR were:

pri-376a2 Fw: 5’-TGGGCTCCGTCGTCATTTT-3’;

pri-376a2 Rv: 5’-CCATCTTTCCACTTACCCTGG-3’;

Q/R Fw: 5’-CCTGGTCAGCAGATTTAGC-3’;

Q/R Rv: 5’-CTAACCTCGCCCATTTTCC-3’.

For the splicing assay, the transfection of SH-SY5Y and HeLa cells with the splicing reporter plasmid, the RNA isolation and the RT-PCR were all performed as described previously.^10^ The primers used for the splicing assay PCR were:

Spl1 (Fw): 5’-TTCTCACTTGGTGGAAGC-3’;

Spl2 (Rv): 5’-CCAGTTGGTAGAGAGAGC-3’.

### Immunoblotting

The cell pellet was resuspended in 15–20 µL of lysis buffer (10 mM Tris-HCl pH 8, 10 mM EDTA pH 8, 0.1 M NaCl, 2% sodium dodecyl sulfate (SDS)) with 1x cOmplete Protease Inhibitor (Roche) and lysed for 30 min at 4°C. The lysate was pelleted by centrifugation and the clear supernatant used for immunoblotting. Protein concentration was measured with Pierce BCA Protein Assay Kit (Thermo Fisher). The lysates were boiled for 5 min in Laemmli buffer prior to electrophoretic separation on an 8% polyacrylamide SDS gel and blotting on a nitrocellulose membrane.

Primary antibodies used were: rabbit anti-FLAG polyclonal antibody (diluted 1:3000; F7425, Sigma-Aldrich), mouse anti-α-tubulin monoclonal antibody (diluted 1:8000; T6074, Sigma-Aldrich). Secondary antibodies used were: horseradish peroxidase (HRP)-conjugated goat anti-rabbit IgG polyclonal antibody (diluted 1:80 000; A0545, Sigma-Aldrich), HRP-conjugated goat anti-mouse IgG polyclonal antibody (diluted 1:5000; A4416, Sigma-Aldrich).

### Immunofluorescence

HeLa cells were seeded and transfected for immunofluorescence as described previously.^10^ Primary antibody staining was performed with rabbit anti-FLAG polyclonal antibody (F7425, Sigma-Aldrich) diluted 1:800 in blocking solution. Secondary antibody staining was performed with Alexa Fluor 568 goat anti-rabbit IgG (H+L) polyclonal secondary antibody (A-11011, Thermo Fisher) diluted 1:200 in phosphate-buffered saline with 0.3 µg/mL 4',6-diamidino-2-phenylindole (DAPI). Samples were analysed with upright microscope Zeiss AxioImager.Z2.

### Data availability

The data supporting the findings of this study are available within the article.

## Results

### Patient evaluations

**Figure 1:**
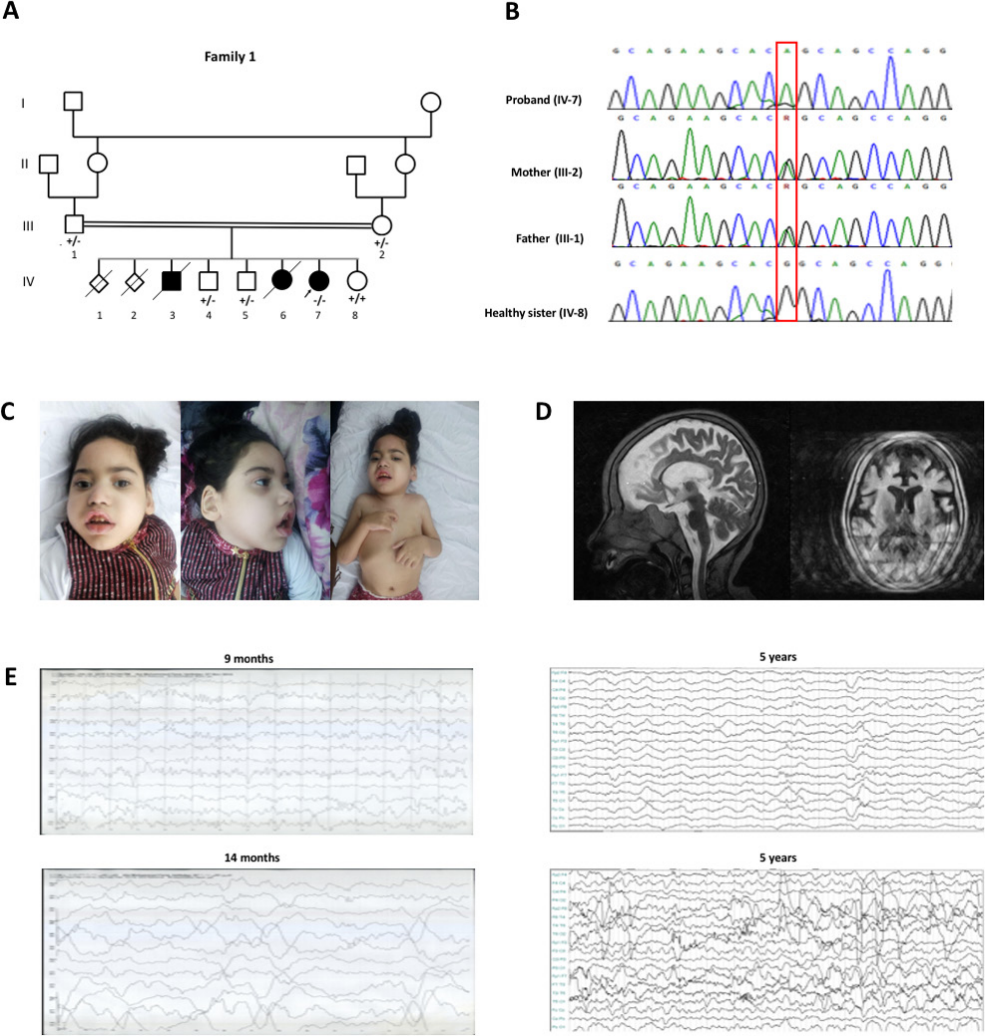
Family 1: pedigree, segregation analysis, chromatograms, clinical photos, brain MRI and electroencephalogram (EEG) screenshots of patient 1. (A) Pedigree showing parental consanguinity, the proband (indicated by the arrow), and two similarly affected siblings (not tested). The genotype of tested individuals is indicated by + (wild-type) and – (mutated). (B) Sanger sequencing chromatograms representative of the segregation of the c.1889G>A, p.(Arg630Gln) variant (NM_015833.4) within the family. (C) Clinical pictures of patient 1 at 5.6 years. Dysmorphic features include sloping forehead, upslanting palpebral fissures, telecanthus, full cheeks, short philtrum, tented upper lip vermilion, pointed chin and pointed and indented helices. Microcephaly, muscle wasting and distal contractures of the upper extremities can also be observed. (D) Brain MRI at 5.6 years (blurred due to involuntary movements) shows diffuse cerebral atrophy with loss of subcortical white matter and enlargement of the ventricular system and the subarachnoid spaces. Corpus callosum hypoplasia is evident in the sagittal T2-weighted section. (E) EEG of patient 1 at different ages. At 9 months, the EEG shows slowing of the background cerebral activity and bilateral focal epileptic discharges. At 14 months, frequent multifocal epileptic discharges may be observed. Eventually, the EEG at 5 years shows recurrent bilateral multifocal epileptiform discharges with secondary generalisation, in the context of a diffuse slowing of the background.

The proband from family 1 (patient 1) is a girl aged 5.6 years born to a consanguineous Iraqi family (figure 1A, B). She had two similarly affected siblings, a male and a female, who died from respiratory infection and distress at the age of 2.5 and 1.5 years, respectively. Family history was remarkable for two miscarriages. The baby was born at 38 weeks’ gestation after an uneventful pregnancy. During the neonatal period, she appeared irritable and excessive crying was observed. At the age of 2 weeks, she experienced infantile spasms consistent with West syndrome and myoclonic jerks, which further progressed to generalised tonic-clonic seizures (GTCS). Seizures occurred >10 times a day, lasting from 30 s to 2 min. Several antiepileptic drugs (AEDs) were tried (including valproic acid, levetiracetam and phenobarbital), but resulted ineffective. Electroencephalogram (EEG) at 9 and 14 months showed bilateral multifocal epileptic discharges in the context of a slowed background activity (figure 1E). Brain MRI at the age of 3 months showed mild cerebral atrophy and white matter abnormalities. Metabolic studies yielded normal results. The patient had a profound DD/ID. At the age of 5 years, she did not have head control and could not sit, even with support. Visual tracking was very poor and she was non-verbal. She had severe microcephaly, with an occipitofrontal circumference (OFC) of 45 cm (−4.3 SD). Neurological examination further revealed strabismus, axial hypotonia combined with appendicular spasticity, lower extremity clonus, diffuse contractures and muscle wasting. Dysmorphic features included epicanthal folds with telecanthus, upslanting palpebral fissures, depressed nasal bridge, short philtrum, tented upper lip, malocclusion with gingival hyperplasia, prominent chin and indented and pointed helices (figure 1C). Brain MRI at the age of 5.6 years revealed diffuse cerebral atrophy, corpus callosum hypoplasia (CCH) and ventricular enlargement (figure 1D). Seizures remained intractable despite multidrug therapy (online supplementary video 1). EEG at 5 years showed frequent bilateral multifocal epileptiform discharges with secondary generalisation in the context of a slow background cerebral activity, suggestive of encephalopathy (figure 1E). She had recurrent respiratory infections, likely due to dysphagia and aspiration. The patient is currently bed-ridden with very limited voluntary movements.

10.1136/jmedgenet-2020-107048.supp1 Supplementary video

**Figure 2:**
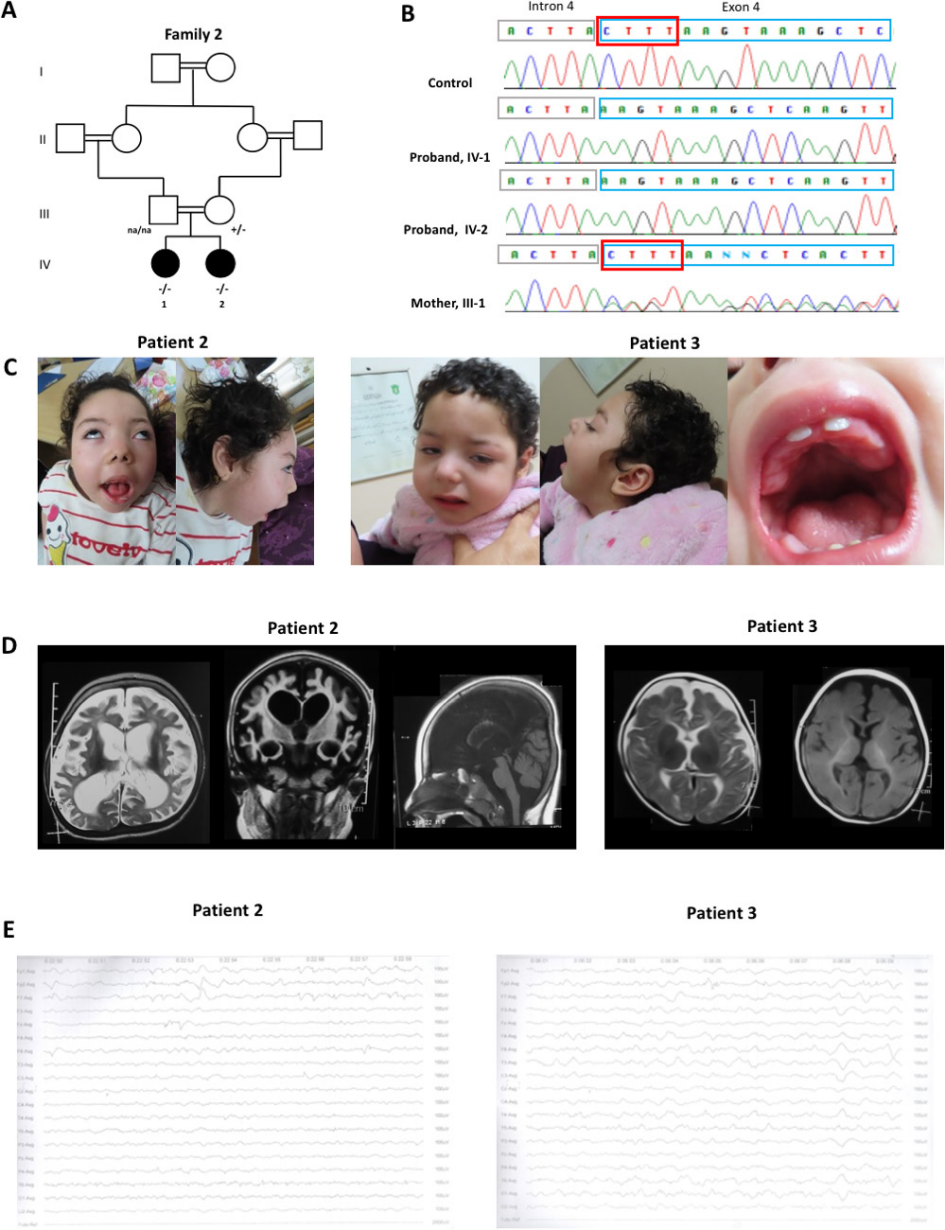
Family 2: pedigree, segregation analysis, chromatograms, clinical photos, brain MRI and electroencephalogram (EEG) screenshots of patients 2 and 3. (A) Pedigree showing multiple consanguinity and the genotypes of tested individuals indicated as + (wild-type) and – (mutated). Paternal segregation was not available (na). (B) Sanger sequencing chromatograms show the segregation of the c.1245_1247+1 del, p.(Leu415PhefsTer14) variant (NM_015833.4) in the two affected siblings (patients 2 and 3) and their mother. The non-coding strand of the *ADARB1* gene is shown. (C) Clinical pictures of patients 2 and 3 at the age of 6 and 4.9 years, respectively. Patient 2 has severe axial hypotonia with lack of head control and strabismus. Her peculiar facial appearance is characterised by sloping forehead, hypertelorism with upslanting palpebral fissures, depressed nasal bridge, triangular nostrils, long and flat philtrum, full cheeks, wide mouth with tented upper lip vermilion and malocclusion, and pointed chin. Her sister, patient 3, is less severely affected and shows milder dysmorphic features which include depressed nasal bridge, full cheeks and long philtrum. In detail, the ogival palate and the gingival hypertrophy in patient 3. (D) Brain MRI of patients 2 and 3 at the age of 4 years and 11 months, respectively. In patient 2, brain MRI shows diffuse cerebral atrophy with relevant loss of white matter and ventricular dilation. Hippocampal atrophy is particularly severe. Linear T2-weighted hyperintensities in the lentiform nuclei can be observed. The T1-weighted sagittal section shows the considerable corpus callosum hypoplasia. In patient 3, cerebral atrophy is predominant in the frontoparietal regions. Subcortical white matter is affected but there is a relative sparing of the basal ganglia. There is hypoplasia of the corpus callosum, but the ventricular enlargement is less severe. (E) EEGs of the affected siblings at the ages of 6 and 4.9 years, respectively. In patient 2, there are slow-wave elements and low-voltage to medium-voltage epileptiform activity predominant in frontal region bilaterally. In patient 3, there are slow, sharp and low to medium-voltage elements in both hemispheres with multifocal origin.

Family 2 consists of two affected sisters aged 6 (patient 2) and 4.9 years (patient 3) from a consanguineous Egyptian family (figure 2A, B). Family history was unremarkable. Both patients were born at term after a regular pregnancy. Neonatal course was uneventful except for irritability and excessive crying. Their psychomotor development was reported normal until the age of 2 and 4 months for patients 2 and 3, respectively. Afterwards, severe DD was diagnosed in both siblings. They had no head control, visual tracking or response to verbal stimuli. The siblings started to experience recurrent seizures at the age of 4–5 months. Their epileptic phenotype was characterised by tonic and myoclonic seizures (including chin myoclonus in patient 2), occurring on a daily basis and lasting for 1–3 min. Medical treatment with several AEDs (including clonazepam, levetiracetam, phenytoin, topiramate, valproate and vigabatrin) resulted ineffective. Neurological examination revealed axial hypotonia, progressive spastic tetraplegia and hyperreflexia in both siblings, although patient 3 was less severely affected. Severe microcephaly was also observed, with an OFC of 43 cm (−5.8 SD) and 43 cm (−5.1 SD) for patients 2 and 3, respectively. Dysmorphic features included high forehead, coarse faces, epicanthal folds, hypertelorism, long flat philtrum, puffy cheeks, open bite with thick alveolar ridge and gingival hyperplasia (figure 2C). Ophthalmological evaluation revealed strabismus and cortical blindness. Auditory brainstem responses were normal. In both patients, seizures remained intractable at the last follow-up examination (online supplementary videos 2 and 3). In patient 2, brain MRI at 4 years showed diffuse cerebral atrophy with relevant subcortical white matter involvement. Additional features were CCH and bilateral T2-weighted abnormalities in the lentiform nucleus. In her sibling, brain MRI at 11 months showed a similar generalised cortical and subcortical atrophy (figure 2D). EEG revealed slow-wave elements and low-voltage to medium-voltage epileptiform activity in both subjects (figure 2E). Extensive metabolic screening including the dosage of organic acid in urine, acylcarnitine profile, ammonia and lactate yielded normal results.

10.1136/jmedgenet-2020-107048.supp2 Supplementary video

10.1136/jmedgenet-2020-107048.supp3 Supplementary video

### Identification of the novel *ADARB1* variants

**Figure 3:**
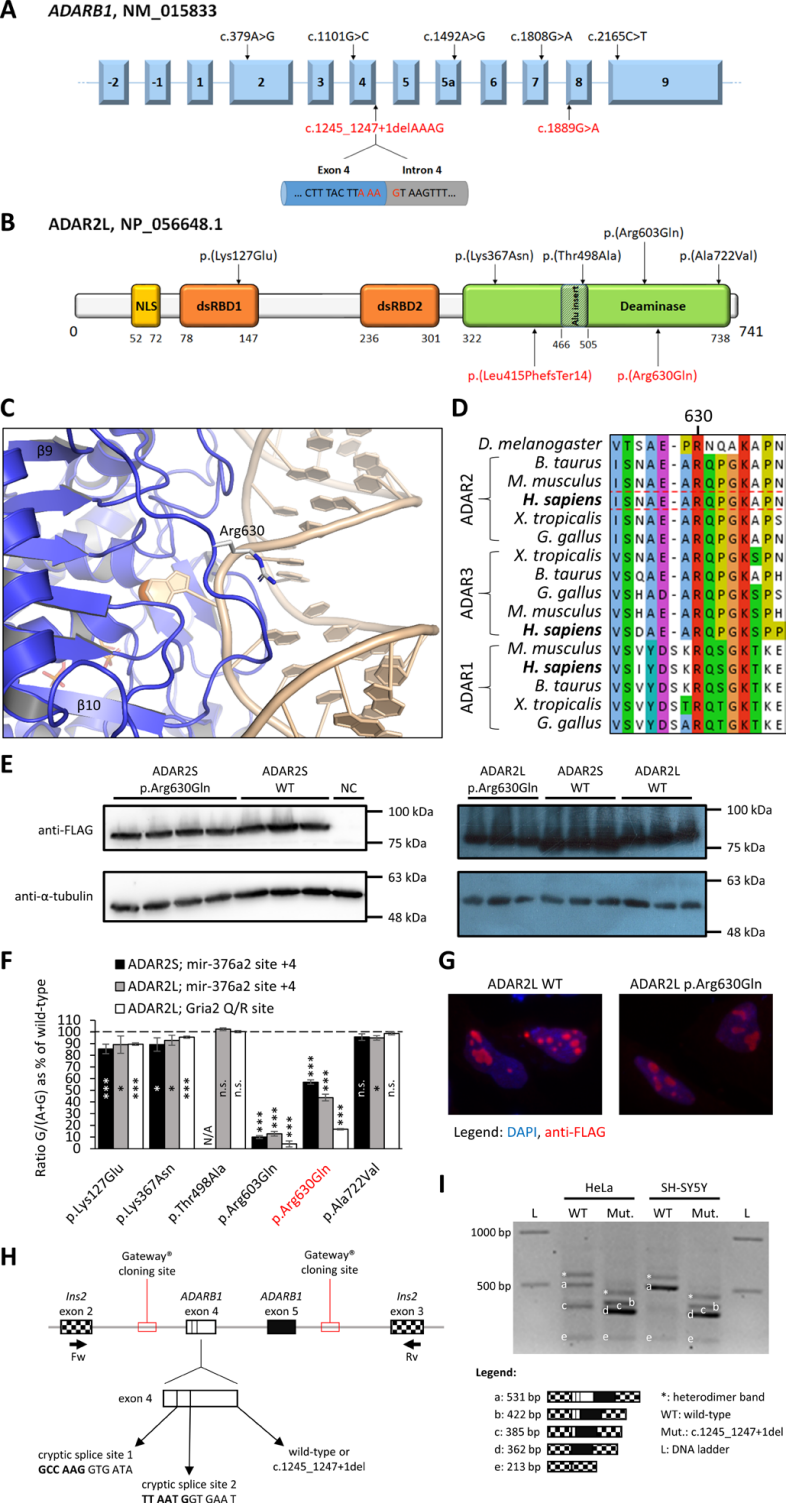
Functional assessment of the ADAR2 variants. (A) Schematic drawing of the NM_015833 transcript of *ADARB1* with the previously reported variants (in black, upside) and the two variants identified in the current study (in red, down). The splicing junction altered by the c.1245_1247+1 del, p.(Leu415PhefsTer14) variant is shown in detail. (B) Cartoon showing the ADAR2 long isoform (ADAR2L) NP_056648.1 with the previously (black, upside) and currently (red, down) reported variants. The *Alu* insertion site is delimited by thin diagonal lines in the context of the deaminase domain. The two RNA-binding domains are indicated as dsRBD1 and dsRBD2. NLS, nuclear localisation signal. (C) Close-up view of a cartoon model of the ADAR2 deaminase domain (blue) with double-stranded RNA (dsRNA) substrate (wheat). Arg630 is drawn as sticks; zinc is shown as an orange sphere, IHP as stick model (PDB ID: 5ED1). (D) Multiple sequence alignment of ADAR1, ADAR2 and ADAR3 from several vertebrate species is shown (human sequence in bold). (E) Immunoblots probed with indicated antibodies showing protein levels of FLAG-tagged ADAR2 wild-type (WT) or p.(Arg630Gln) after co-transfection of HEK 293T with plasmids expressing ADAR2 and *pri-mir-376a2*. Lanes with the same labels represent replicates. NC, non-transfected control. (F) Graph showing editing of two tested substrates by ADAR2S or ADAR2L proteins in transiently transfected HEK 293T. Previously tested variants in black,^10^ new tested variant in red. Ratio G/(A+G) is the ratio of the guanosine peak height to the sum of adenosine and guanosine peak heights of the sequencing chromatograms. Editing levels were normalised to the editing by the WT protein, which is set as 100% (indicated by dashed line). Data represent means±SD (n≥3 independent experiments). *P≤0.05, ***p≤0.001. N/A, not available; n.s., not significant. (G) HeLa cells were transiently transfected with plasmids expressing the indicated FLAG-tagged proteins and analysed by indirect immunofluorescence. Cells were probed with anti-FLAG antibody (red channel) with DAPI (blue channel) used as a DNA stain. Cells with representative staining pattern are displayed. (H) Schematic drawing of part of the splicing reporter plasmid. *ADARB1* exon 4 with two cryptic splice sites is shown in detail. *Ins2* stands for rat insulin-2 gene. Exons are shown as boxes, introns as lines. The positions of primers used for PCR are indicated below. (I) Electrophoretogram of splicing products from HeLa and SH-SY5Y cells transiently transfected with WT or c.1245_1247+1 del splicing reporter plasmid. Each band was cut out from the gel and its identity was confirmed by Sanger sequencing. Reverse transcription was performed twice, each time with a different primer (either oligo(dT)_18_ or a transcript-specific primer Spl2). Experiment was performed with biological triplicates, and a representative agarose gel (with oligo(dT)_18_ used for reverse transcription) is shown.

In Family 1, the novel homozygous missense variant c.1889G>A, p.(Arg630Gln) in exon 8 of *ADARB1* was detected (chr21:45 220 857 G>A, GRCh38) (figure 3A). This variant is located within a ~37 Mb region of homozygosity and is absent in publicly available population databases as well as our in-house database. The p.(Arg630Gln) variant occurs at a highly conserved amino acid residue (GERP: 4.59, CADD: 29.4; figure 3D), and segregated with the disease in the family. In silico analysis predicts that the missense variant p.(Arg630Gln) is deleterious (CADD: 29.4; FATHMM: Pathogenic (0.872061); MutationAssessor: Medium (2.35); MutationTaster: Disease causing (D 0.99); PolyPhen-2: Probably damaging (1.00); PROVEAN: Deleterious (D 0.02); SIFT: Damaging). The affected residue is located in the deaminase domain of ADAR2 (figure 3B). Arg630 lies in the middle of the β9-β10 loop (figure 3C).^14^ This loop partially extends across the RNA-binding surface of the deaminase domain, and Arg630 points towards the major groove of the dsRNA substrate. Arg630 is highly conserved in all three ADAR family members in vertebrates (figure 3D).

In Family 2, ES led to the identification of the novel homozygous deletion c.1245_1247+1 del, p.(Leu415PhefsTer14) (chr21:45 182 751delAAAG, GRCh38) in exon 4 and the downstream intron of *ADARB1* (figure 3A). This 4-bp deletion affects the donor splice site in *ADARB1* intron 4 (figure 3A) and is predicted to result in incorrect splicing of the affected intron.

### Impaired ADAR2 editing activity caused by p.(Arg630Gln)

To investigate the functional significance of the ADAR2 variants, an in vitro RNA editing assay was performed, as suggested by the very recently published paper by Tan *et al*.^10^ To assess the effects of the p.(Arg630Gln) variant on RNA editing activity, we co-transfected the plasmid expressing the FLAG-tagged ADAR2 variant and the plasmid expressing a known editing substrate (either human *pri-mir-376a2* or murine *Gria2* Q/R site) into HEK 293 T cells for the RNA editing assay.^15 16^ The effects of the variant were tested in each of the two major isoforms of ADAR2 protein, ADAR2S (ADAR2a; UniProt ID: P78563-2) and ADAR2L (ADAR2b; UniProt ID: P78563-1). To ensure ADAR2 protein levels were approximately the same in all samples, we performed immunoblotting with whole cell lysates from transfected HEK 293T and probed the membranes with anti-FLAG antibody (figure 3E). Next, total RNA from transfected cells was used for RT-PCR and Sanger sequencing. With both the assayed editing substrates, we detected a large decrease in editing activity of the ADAR2 p.(Arg630Gln) variants compared with the respective wild-type proteins (figure 3F).

### p.(Arg630Gln) does not affect ADAR2 subcellular localisation

We further analysed the subcellular localisation of ADAR2 p.(Arg630Gln). To this end, transiently transfected HeLa cells were used for indirect immunofluorescence. We did not observe any effect of the p.(Arg630Gln) variant on localisation of ADAR2 as both wild-type and mutant proteins showed the same localisation pattern (figure 3G). Consistent with previous reports, ADAR2 localised to the nucleus and accumulated in the nucleoli.^17 18^

### Impaired *ADARB1* splicing caused by c.1245_1247+1del

Next, a minigene encompassing the region around *ADARB1* exon 4 and 5 was cloned into pDESTsplice splicing reporter plasmid and PCR mutagenesis was used to introduce the c.1245_1247+1 del variant (figure 3H).^13^ This construct was then transiently transfected into SH-SY5Y (metastatic neuroblastoma) and HeLa (cervical cancer) cells. One day after the transfection, total RNA was isolated, DNase treated and RT-PCR was performed to assess potential splicing defects.

The wild-type construct was correctly spliced in SH-SY5Y cells, whereas in HeLa cells a fraction of transcripts was incorrectly spliced due to recognition of the cryptic splice site 1 (figure 3H, I). This difference is possibly a reflection of the different origin of SH-SY5Y (neuronal) and HeLa (epithelial) cells. The introduction of c.1245_1247+1 del variant into the minigene lead primarily to exon 4 skipping in both cell lines. Additionally, two cryptic splice sites inside exon 4 were used in a fraction of transcripts originating from the c.1245_1247+1 del minigene in both cell lines (figure 3H, I). Regardless of the splicing outcome, the effect on translation is similar, as exon 4 skipping or use of either of the cryptic splice sites causes a frameshift with a premature stop codon in *ADARB1* mRNA. We conclude that the c.1245_1247+1 del mRNA cannot encode full-length ADAR2 protein and it is most likely targeted for nonsense-mediated decay (NMD).

## Discussion

We have identified two novel biallelic variants in *ADARB1*, a missense and a deletion, segregating in three individuals with a severe DEE from two unrelated families. Both of these very rare variants result in a loss of function of ADAR2, supporting this as the pathogenic mechanism underlying *ADARB1*-related DEE.

These results are in line with an autosomal recessive pattern of inheritance that has been observed in *Adar2* null mice. In fact, studies in mice elucidating some functions of ADAR2 in brain physiology revealed that *Adar2* knockout mice die due to seizures within 3 weeks after birth, but a single-allele knockout of *Adar2* is still viable. Similarly, heterozygous *ADARB1* variants did not lead to epilepsy in the subjects of the above-mentioned study, whereas biallelic variants resulted in a severe encephalopathy.^10^ In agreement with these observations, the loss-of-function observed/expected upper bound fraction is 0.4 and *ADARB1* is predicted to be likely associated with a recessive rather than a dominant disorder by DOMINO.^19^

The main molecular pathogenic mechanism underlying *ADARB1*-related encephalopathy is most likely represented by the ADAR2-mediated recoding of brain pre-mRNAs. In the brain, ADAR2-mediated A-to-I editing recodes the transcripts encoding glutamate (eg, *GRIA2*, *GRIK1*) and serotonin (eg, *HTR2C*) receptor subunits, modulating their functions.^1 7 8^ Consequently, deregulation of A-to-I editing of these transcripts has been associated with a wide range of neurological and psychiatric disorders.^20 21^ Of note, the viability of *Adar2* null mice is restored on insertion of the pre-edited *Gria2 R* sequence into the mouse genome.^22^ The same lethal phenotype was observed in mice with the editing-incompetent allele of *Gria2*.^9^ This indicates that the lethality in mice is exclusively mediated by the lack of editing of the *Gria2* Q/R site. GRIA2 is a subunit of the ionotropic α-amino-3-hydroxy-5-methyl-4-isoxazolepropionic acid (AMPA) type of glutamate receptor and the ADAR2 editing determines the AMPA receptor calcium permeability. In humans, heterozygous *GRIA2* variants were identified in 28 unrelated individuals with DEE.^23^ These observations are consistent with our hypothesis that the deleterious *ADARB1* variants manifest via *GRIA2* Q/R site under-editing.

The under-editing of GRIA2 in *ADARB1* patients suggest that the early use of the recently developed drug perampanel (a selective and non-competitive antagonist of the AMPA receptor) might be beneficial in controlling the seizures. In a small study with a mouse model of sporadic amyotrophic lateral sclerosis (sALS), perampanel administration successfully prevented the progressive loss of motor neurons caused by the conditional knockout of *Adar2* and the subsequent excitotoxicity due to *Gria2* under-editing.^24^ The clinical trials of perampanel efficacy in treatment of patients with sALS are ongoing.^25^ This drug is currently approved for the treatment of intractable epilepsy and could allow a better seizure control in *ADARB1* patients. Furthermore, an early intervention on the epilepsy could modify the natural history of this condition, possibly decreasing or slowing the progression of the encephalopathic changes.^26^

In this report, we expand the phenotypic characterisation of a new form of severe DEE caused by biallelic variants in *ADARB1*. This condition is characterised by profound DD, intractable epilepsy, spasticity, and cerebral atrophy (table 1).

**Table 1:** Clinical characteristics of the subjects with *ADARB1* variants

	Family 1 (Iraq)	Family 2 (Egypt)		Tan *et al* 10 (Caucasian, Hispanic, Azari)
	Pt 1 (IV-7)	Pt 2 (III-1)	Pt 3 (III-2)	4 pts, 4 families
Age at last FU, sex	5.6 y, F	6 y, F	4.9 y, F	Mean 5.2 y, 4 M
Alive	+	+	+	+ (3), one died at 2 y
Consanguinity	+	+	+	+ (2)
Previous miscarriages	+	–	–	+ (1)
Similarly affected siblings	+ (2, deceased)	+ (III-2)	+ (III-1)	–
Pregnancy	Regular (38 we)	Regular (40 we)	Regular (38 we)	Regular (3), pre-eclampsia (1)
Birth complications	–	–	–	–
OFC at birth	N/A	33.3 cm (−0.72 SD)	33 cm (−0.7 SD)	0.38 to −2.2 SD (2 N/A)
Neonatal course				
Irritability	+	+	+	N/A
Excessive crying	+	+	+	N/A
Developmental history				
Visual tracking	Poor	–	–	– (3)
Head control	–	–	–	– (3)
Sit with support	–	–	–	– (3)
Standing with support	–	–	–	– (3)
Walking with support	–	–	–	– (4)
Speech	–	–	–	– (3), few words (1)
Intellectual disability	Profound	Profound	Profound	Profound (2), severe (2)
Feeding difficulties	+	+	+	+ (4)
Dysmorphic features	+	+	+	+ (2)
OFC at last FU	45 cm (−4.3 SD)	43 cm (−5.8 SD)	43 cm (−5.1 SD)	−3.3 to −4.4 SD (mean −3.8 SD)
Neurological features				
Axial hypotonia	+	+	+	+ (4)
Spastic tetraplegia	+	+	+	+ (2)
Hyperreflexia	+	+	+	+ (1)
Sleep disturbance	+	–	–	+ (1)
Other	Bruxism, insomnia	–	–	Tremor (1), staring spells (1), repetitive movements (1)
Vision				
Strabismus	+	+	+	+ (1)
Other	–	Cortical blindness	Cortical blindness	Cortical blindness (3)
ABRs	N/A	Normal	Normal	Normal (4)
Epilepsy				
Onset	15 d	4 mo	5 mo	2–7 mo (mean 4.3)
Type	MCS, GTCS, IS	TS, MCS	TS, MCS	FS, TS, GS, GTCS
Frequency, duration	Daily, 0.5–2 min	Daily, 1–3 min	Daily, 1–3 min	Weekly to daily
Associated signs	Apnoea, staring	Head deviation, vomiting	Head deviation	Eye deviation, twitching, apnoea
EEG	MFDs, slow background	MFDs	Bilateral TPDs	MFDs, slow background
Status epilepticus	–	–	–	+ (2)
Response to AEDs*	–	–	–	– (4)
Evolution	GTCS (LGS)	TS, MCS	TS, MCS	GTCS
Current status	Intractable	Intractable	Intractable	Intractable
Neuroimaging features†				
Diffuse cerebral atrophy	+	+	+	+ (3), temporal lobes (1)
White matter loss	+	+	+	+ (2)
Delayed myelination	–	+	+	+ (2)
CCH	+	+	+	+ (3)
Enlarged ventricles	+	+	+	+ (3)
Basal ganglia T2-weighted hyperintensity	–	+	–	- (3)
Other features	Contractures, muscle wasting, 2 hypopigmented spots on the sternum	PDA	–	Laryngomalacia (1), plagiocephaly (2), cryptorchidism (1), contractures and muscle wasting (1)
Metabolic investigations‡	Normal	Normal	Normal	Normal (4)

*AEDs included clonazepam, levetiracetam, phenytoin, topiramate, valproate and vigabatrin.

†MRI pictures of three out of four patients were available for review.

‡Extended metabolic screening including organic acid in urine, acylcarnitine profile, ammonia and lactate.

ABRs, auditory brain responses; AEDs, antiepileptic drugs; CCH, corpus callosum hypoplasia; d, days; EEG, electroencephalogram; F, female; FS, focal seizures; FU, follow-up; GS, generalised seizures; GTCS, generalised tonic-clonic seizures; IS, infantile spasms; LGS, Lennox-Gastaut syndrome; M, male; MCS, myoclonic seizures; MFDs, multifocal discharges; mo, months; N/A, not available; OFCS, occipitofrontal circumference; PDA, patent ductus arteriosus; Pts, patients; s, syndrome; TPDs, temporoparietal discharges; TS, tonic seizures; we, weeks; y, years.

At birth, affected children appear healthy and congenital microcephaly has been observed in only one case.^10^ Excessive irritability and crying, and feeding difficulties occur in the first months of life. Afterwards, a profound DD becomes evident as most subjects lack head control, cannot sit even with support and are non-verbal. Epilepsy starts in the first year of life, especially in the first semester. Seizure types include focal, myoclonic, tonic and GTCS. In our cohort, patient 1 also showed infantile spasms leading to a diagnosis of West syndrome. The response to AEDs is very poor with recurrent and intractable seizures despite multidrug treatment. EEG features consist of slowing of background cerebral activity and multifocal epileptic discharges. Seizure evolution is variable, but many patients develop intractable GTCS. Status epilepticus has been reported in two previous patients by Tan *et al*.^10^ Affected individuals develop progressive microcephaly (up to −5.8 SD) and spastic tetraplegia, with very limited voluntary movements. A specific facial gestalt cannot be recognised, but some dysmorphic features (eg, apparent hypertelorism, upslanting palpebral fissures, depressed nasal bridge and pointed chin) recur. All our patients showed gingival hypertrophy, but this finding might be attributed to phenytoin treatment. While hearing is usually spared, strabismus and cortical blindness are common.

As to the neuroimaging features, all affected individuals showed a variable combination of severe structural changes of the CNS. In line with the very recent report by Tan *et al*,^10^ delayed myelination and diffuse cerebral atrophy with relevant white matter involvement were observed in our cases. Furthermore, brain MRI revealed a considerable CCH. Peculiar T2-weighted hyperintensities were identified in the lentiform nuclei of patient 2. Of note, basal ganglia may also be spared in some cases, as suggested by the lack of evident atrophic changes in the putamen and globus pallidus in patient 3. However, the milder abnormalities observed in this case likely reflect the younger age at MRI, as the disease course is progressive. The ongoing monitoring of affected individuals will help clarify the evolution of the atrophic process over time and the peculiar involvement of the diverse cerebral structures. Although non-specific, these neuroimaging abnormalities illustrate the severity of this condition and represent useful clues to support the electro-clinical diagnosis.

A remarkable feature of *ADARB1*-related DEE is the variable expressivity. Although this condition is generally severe (table 1), one of the patients very recently reported by Tan *et al* (individual 1) was able to stand with support and showed a milder epileptic phenotype, with staring spells and few GTCS episodes.^10^ Fibroblasts from this patient appeared to express more ADAR2 than control fibroblasts and the editing activity of ADAR2 variants from this patient was less affected compared with the rest of the cohort. Although this subject had profound DD, microcephaly and severe feeding difficulties, his electroclinical features were milder than all other known *ADARB1* cases. Similar observations arise on the neuroimaging aspects, as white matter involvement was less severe in the individuals 3 and 4 from Tan *et al*.^10^ Future studies and the identification of further cases will help better define the phenotypic spectrum and delineate the neuroradiological aspects of this complex disorder.

The variants reported in this study expand and complement the spectrum of deleterious *ADARB1* variants.^10^ The p.(Arg630Gln) variant observed in patient 1 affects a highly conserved positively charged residue at the RNA-binding surface of the ADAR2 deaminase domain (figure 3C).^14^ The positive charge of the RNA-binding surface is necessary for the interaction of ADARs with the negatively charged dsRNA substrate backbone. Thus, this variant is likely to disrupt the protein-RNA interface, which is consistent with the strong decrease observed in the RNA editing activity. Furthermore, arecently published study revealed that Arg630 (corresponding to Arg590 in ADAR2Sisoform) plays an important role in mediating protein-protein interactions inthe asymmetric dimer of ADAR2.^27^ Therefore, the p.(Arg630Gln) variant probably has anegative impact on ADAR2 dimerization, which is required for efficient editingof some ADAR2 substrates. Other functionally relevant protein-protein interactions of ADAR2 couldbe affected by this variant as well.^28–32^ The c.1245_1247+1 del variant causes incorrect splicing of *ADARB1* minigene transcripts due to exon skipping and cryptic splice site activation. Hence, we expect that *ADARB1* mRNAs from patients 2 and 3 all contain a frameshift and a premature stop codon. Such mRNAs are most likely targeted for NMD.

In conclusion, we contribute to the delineation of *ADARB1*-related DEE phenotype, stressing the progressive course of this condition and the most relevant clinical aspects (profound DD/ID, progressive microcephaly and spastic tetraplegia and intractable epilepsy). The identification of the null c.1245_1247+1 del variant and the functional characterisation of the c.1889G>A variant supported a loss-of-function pathogenic model. In conclusion, these data show the pathogenic role of biallelic *ADARB1* variants as the cause of a new distinctive DEE in the group of RNA editing-related disorders, reinforcing the importance of RNA editing in brain function and development. Our study further suggests that *ADARB1* variants should be screened in DEE cases tested with ES and that *ADARB1* should be included in the epileptic encephalopathies next-generation sequencing panels.

## Data Availability

The data supporting the findings of this study are available within the article.
